# Profiles of total worker health® in United States small businesses

**DOI:** 10.1186/s12889-021-11045-8

**Published:** 2021-05-29

**Authors:** Natalie V. Schwatka, Miranda Dally, Erin Shore, Lynn Dexter, Liliana Tenney, Carol E. Brown, Lee S. Newman

**Affiliations:** 1 Center for Health, Work & Environment, Colorado School of Public Health, University of Colorado, Anschutz Medical Campus, 13001 E. 17th Pl., 3rd Floor, Mail Stop B119 HSC, Aurora, CO 80045 USA; 2 Department of Environmental and Occupational Health, Colorado School of Public Health, University of Colorado, Anschutz Medical Campus, 13001 E. 17th Pl., 3rd Floor, Mail Stop B119 HSC, Aurora, CO 80045 USA; 3 Department of Epidemiology, Colorado School of Public Health, University of Colorado, Anschutz Medical Campus, 13001 E. 17th Pl., 3rd Floor, Mail Stop B119 HSC, Aurora, CO 80045 USA

**Keywords:** Safety climate, Health climate, Safety leadership, Health promoting leadership, Health leadership, Occupational safety and health, Safety behavior, Health behavior, Latent profile analysis

## Abstract

**Background:**

The Total Worker Health® (TWH) approach is a best practice method to protect and promote worker safety, health, and well-being. Central to this approach is leadership support and health and safety climates that support day-to-day use of health and safety policies and programs. There is some research that supports these relationships, but there is limited research amongst small businesses. Furthermore, it remains to be shown what role TWH business strategies, as reflected by organizational policies and programs, play in this process. The purpose of this study is to characterize small businesses by their organizations’ TWH approach and assess the relationship of these approaches to employee health and safety behaviors.

**Methods:**

We utilized cross-sectional data from 97 businesses participating in the Small+Safe+Well study. We collected data using a business assessment tool, Healthy Workplace Assessment™, and an employee assessment tool, Employee Health and Safety Culture Survey. We used latent profile analysis at the business level to identify subgroups of businesses based on a set of characteristics from these assessments. Linear regression analysis at the employee level was used to determine profile association with employee safety and health behaviors.

**Results:**

There were two profiles characterized by the lowest (33% of all businesses) and highest (9%) levels of the indicators. There were also two profiles with higher scores on two of the different foci on either TWH business strategies (27%) or leadership and climate (31%). Employees working for a business with a profile that focused on leadership and climate, in addition to having a business strategy, reported the best safety and health behaviors.

**Conclusions:**

Our study demonstrates that employee engagement in TWH will be highest when businesses have a strategy for how they implement a TWH approach and when they demonstrate leadership commitment to these strategies and foster positive safety and health climates. Our results offer suggestions on how to use TWH assessments to develop interventions for small businesses. More research is needed to understand whether small businesses can improve upon their profile overtime, whether these changes depend on contextual factors, and whether TWH interventions can help them improve their profile.

**Supplementary Information:**

The online version contains supplementary material available at 10.1186/s12889-021-11045-8.

## Background

The Total Worker Health® (TWH) approach is emerging as a best practice method to protect and promote worker safety, health, and well-being. Businesses can encounter numerous obstacles in the pursuit of TWH [1]. That is why leadership support and the creation of health and safety climates that that support day-to-day engagement in health and safety policies and programs are central to the TWH approach [2, 3]. This is because employees will be more likely to participate in efforts to protect and promote their health if they work in an environment that cares about their health [4, 5]. While some research supports the relationship between leadership support, safety climate and health climate, and employee participation, there has been limited research amongst small businesses. Furthermore, it remains to be shown what role TWH business strategies, the organizational policies and programs to protect and promote employee health, play in the relationship between between leadership support, safety climate and health climate, and employee participation.

It is important to conduct this research in small enterprises because, globally, the majority of people work for small businesses and suffer a significant amount of work-related injuries, illnesses, and fatalities as well as poor health [6]. We find that small businesses are implementing a variety of TWH business strategies to protect and promote employee health [7], but employees of small businesses report varying levels of their employers’ commitment to the TWH business strategies [8]. There is a need to simultaneously characterize what small employers are doing, both in terms of business strategies as well as leadership and climate, to protect and promote their employees’ health. This information can inform the design and implementation of needed small business TWH interventions [9].

According to the theory of social exchange, when organizations provide employees with resources to protect and promote their health, employees will be motivated to reciprocate by engaging in safety and health behaviors [10]. These resources can take the form of business strategies as well as indicators of leadership and climate. We define leadership to be a commitment to safety and health via communication, role modeling, positive feedback, resource allocation, and accountability. While climate reflects employee perceptions that their organization is committed to their safety and health. Meta-analyses support the positive relationship between health and safety leadership, climate, and behavior [4, 5]. However, there is little research that describes the relationship between health and safety business strategies and employee behavior [8]. Each of these three types of resources - business strategies, leadership, and climate – contributes information about the work environment as it relates to TWH. Business strategies reflect information about the existence of policies and programs that protect and promote employee health, whereas ratings of leadership and climate provide us with an indication of whether the business strategies are implemented successfully. Employee engagement in worksite health and safety practices is critical to the success of any TWH program [11]. However, an understanding of the characteristics of small businesses that elicit health and safety behaviors is limited.

The purpose of this study is to characterize small businesses by their organizations’ TWH approach -- business strategies, leadership, and climate; and to assess the relationship of these approaches to employee health and safety behaviors. Using latent profile analysis, we hypothesized that small business could be characterized by indicators of TWH business strategies as well as employee perceptions of leadership commitment safety, leadership commitment to health, safety climate, and health climate. Specifically, we hypothesized (H1) that there would be four profiles: 1) A beginner profile with the lowest indicator scores, 2) A business strategy-focused profile where scores on the TWH business strategy indicators were high but scores on climate and leadership indicators were low, 3) A culture-focused profile where scores on climate and leadership indicators were high but scores on TWH business strategy indicators were low, and 4) An advanced profile where scores on all indicators were high. The culture-focused profile was named as such because leadership and climate indicators have been associated with a culture of health and safety in prior literature [12]. On the other hand, the business strategy profile was named as such because of its focus on TWH policies and programs. We also hypothesized (H2) that employees who worked for businesses with higher TWH business profile scores would report better safety and health behaviors than employees who worked for businesses with lower TWH business profiles scores.

## Methods

### Sample

The businesses in this study are part of the Small+Safe+Well (SSWell) intervention study, which seeks to test whether a small business (< 500 employees) TWH intervention influences TWH policies, procedures, and practices, safety and health climates and worker health [2]. In total, we recruited 97 businesses from April 2017 through September 2019 through email marketing, regional events, and channel partners including chambers of commerce, workers’ compensation insurers, local public health agencies, health and wellness coalitions, and trade associations. Once a business enrolled in the study, they were invited to take the Health Links Healthy Workplace Assessment™. Upon completion of this assessment, employees from participating organizations were invited to participate in the Employee Health and Safety Culture Survey. The study coordinator generated a unique survey link and sent it along with a recruitment email to our main contact at each organization, who then forwarded the link to their employees. The online survey was available for 2 weeks and the study coordinator sent a reminder email to the main contact half-way through that period. Employees who completed the survey had the option to enter their email address into a raffle to win one of 15 $100 gift cards. Email addresses were collected on a separate database. No identifying information was collected in the Employee Health and Safety Culture Survey, and the employer was blinded to the individual level responses and to whether employees completed the survey.

To test our hypotheses, we utilized cross-sectional data from the Healthy Workplace Assessment and cross-sectional data from the Employee Health and Safety Culture Survey completed by employees in the participating 97 businesses before they participated in the intervention. This study was approved by the Colorado Multiple Institutional Review Board (COMIRB) and informed consent was obtained from all participants. All methods were carried out in accordance with relevant guidelines and regulations.

### Measures

#### Business assessment

The Health Links Healthy Workplace Assessment is a web-based instrument that is completed by one representative in consultation with others in the organization best familiar with the business’s TWH strategy. The respondent represented individuals in leadership, management, human resources, health and safety, and administration. Our prior research demonstrates that responses to each of the questions are not affected by who completed the survey [7]. It includes 35-items that all refer to the previous 12 months and are answered with a “yes” or “no” response. The assessment measures existing evidence-based TWH strategies across 6 core benchmarks including organizational supports (30 max points), workplace assessment (12 max points), health policies and programs (16 max points), safety policies and programs (16 max points), engagement (16 max points), and evaluation (10 max points) [7]. All questions can be viewed on the Health Links website: https://www.healthlinkscertified.org/get-started.

#### Employee assessment

The Employee Health and Safety Culture Survey contained 109 items that asked about employees’ demographics as well as their perspectives on several constructs addressing organizational environment in general as well as workplace health and safety. In the present study, we evaluated the questions pertaining to leadership commitment, climate, and behavior. We developed the leadership commitment to safety (5-items) and leadership commitment to worksite wellness (5-items) items to reflect leaders’ communication, role modeling, employee recognition, resource allocation, and accountability. For safety climate, we used Lee et al.’s [13] organizational commitment to safety measure (5-items). For health climate, we used Zweber et al.’s [14] organizational commitment to health measure (4-items). Finally, safety behaviors (3-items) and health behaviors (3-items) were measured by adapting Griffin and Neal’s [15] safety participation scale. These measures have been found to be reliable and valid in prior research [8, 16, 17].

### Analysis

We employed a latent profile analysis analytical technique to identify subgroups of businesses based on a set of characteristics. As a person-centered approach to analysis (as opposed to variable-centered approach), it allows us to consider the whole occupational environment and the combined impact of certain characteristics rather than focusing on the impact of one variable at a time [18]. It has been used to categorize workplace healthy leadership [19], working conditions [20], organizational health promotion practices [21], and the co-occurrence of workplace health protection and promotion practices [22]. However, to our knowledge, it has not been used to simultaneously understand the working environment in terms of both the business strategy and leadership and climate for health and safety.

We addressed hypothesis 1 by conducting a latent profile analysis at the business level. For the variables measured at the employee level, we calculated the ICC (1) and r*wgj prior to aggregating responses to the business level. We used the ICC (1) estimate to determine whether there was sufficient evidence that business membership influenced individual ratings (sufficient variance between and within businesses) to aggregate responses at the business level. We also calculated r*wgj to determine whether there was sufficient agreement in responses (interchangeability) to aggregate responses at the business level [23]. The estimates for both the ICC (1) and r*wgj indicated sufficient evidence that the variables measured at the employee level could be aggregated at the business level (see Additional file 1). We then specified a series of latent profile models, starting with two, and increasing the number of latent profiles until the model fit no longer improved.

We used several fit statistics to evaluate the fit of each of the latent profile models: log likelihood (LL – the best model has the lowest score), Akaike information criterion (AIC – the best model has the lowest score), Bayesian information criterion (BIC – the best model has the lowest score) sample-size-adjusted BIC (the best model has the lowest score), Lo-Mendell-Rubin likelihood ratio test (LMR – significant test at *p* < 0.05), bootstrap likelihood ratio test (BLRT – significant test at *p <* 0.05), and entropy (1 indicates the profiles are perfectly separated). We also obtained the average latent profile probabilities for most likely latent profile membership (i.e., the probability that the business belongs to their specified profile) and the final profile counts for the latent profile based on their most likely latent profile membership (i.e., the number of businesses per profile). We chose the final model based on these fit statistics as well as the meaningfulness of the solution. All analyses were completed in MPlus 8 Version 1.6 [24].

Next, we addressed hypothesis 2 with a linear regression analysis at the employee level. Each employee was assigned a TWH business profile based on what profile their business fell into in the LPA analysis described above. Two models were estimated. First, the effect of latent profile membership on safety behaviors and second on health behaviors. The models controlled for industry, number of employees, whether they were in a management role, tenure, age, and gender. The models also included a random intercept for businesses to account for variability in the outcome by business. These analyses were completed in Stata Version 14.2 [25].

## Results

### Latent profile analysis

**Fig. 1:**
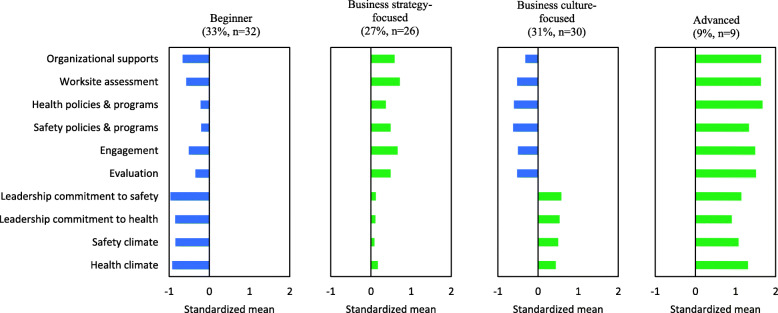
Standardized means of each of the indicators for the final four latent profile solution. Green bars reflect better than average scores and blue bars reflect worse than average scores

Latent profile solutions emerged for two, three, and four profiles. Convergence failed with a fifth profile solution. Based on our selection criteria, the four latent profile solution fit the data the best and the probabilities that businesses would be assigned to their profile were high (> 0.90), indicating a low chance of misclassification (see Additional file 1). There were two profiles characterized by low and high levels of the indicators as well as profiles with two different foci on either TWH business strategies or leadership and climate. Table 1 presents the mean indicator scores for each of the four profiles and Fig. 1 displays these results as standardized means, which have been rescaled to have a mean of zero and a standard deviation of one. Table 2 includes the business demographics for each profile and demonstrates that profile membership did not depend on the number of employees or industry at the *p* < 0.01 level.

**Table 1:** The four latent profile solution’s mean scores on each indicator (*n* = 97)

	Maximum possible score	Profile 1: Beginner (*n* = 32)	Profile 2: Business strategy- focused (*n* = 26)	Profile 3: Culture- focused (*n* = 30)	Profile 4: Advanced (*n =* 9)
Organizational supports	30	11.01	19.14	13.38	26.27
Worksite assessment	12	1.29	4.75	1.43	7.53
Health policies & programs	16	2.72	4.52	1.70	8.21
Safety policies & programs	16	8.92	11.27	7.25	14.39
Engagement	16	5.75	8.73	5.77	10.91
Evaluation	10	2.35	3.87	1.98	5.80
Leadership commitment to safety	5	3.26	3.69	3.88	4.11
Leadership commitment to health	5	3.19	3.61	3.79	3.97
Safety climate	5	3.40	3.85	3.96	4.32
Health climate	5	3.57	3.95	4.11	4.36

**Table 2:** Number of businesses, employees, and industries represented by final latent profile solution (*n* = 97)

	Profile 1: Beginner	Profile 2: Business strategy-focused	Profile 3: Culture-focused	Profile 4: Advanced
Number of businesses (n)	32	26	30	9
Number of employees (M (SD))^a^	74 (61)	117 (127)	49 (61)	89 (73)
Industry (n (%))^b^				
Agriculture	0 (0%)	0 (0%)	1 (3%)	0 (0%)
Construction	0 (0%)	3 (12%)	3 (10%)	1 (11%)
Health Care & Social Assistance	11 (35%)	6 (23%)	5 (17%)	2 (22%)
Manufacturing	2 (6%)	2 (8%)	5 (17%)	1 (11%)
Mining/oil	0 (0%)	0 (0%)	0 (0%)	1 (11%)
Public admin	3 (10%)	5 (19%)	2 (7%)	1 (11%)
Real estate	1 (3%)	1 (4%)	2 (7%)	0 (0%)
Retail/Wholesale Trade	0 (0%)	1 (4%)	0 (0%)	1 (11%)
Services	14 (45%)	6 (23%)	11 (37%)	2 (22%)
Transportation, warehouse	0 (0%)	2 (8%)	1 (3%)	0 (0%)

^a^One-way ANOVA (*F*(3,93) = 2.69, *p* = 0.05

^b^χ^2^ (27, *N* = 96) = 32.38, *p* = 0.22

#### The beginner profile

The businesses in the beginner profile represented 33% of all businesses in the sample. Businesses in this profile generally had the lowest scores on all indicators, except for health policies and programs, safety policies and programs, and evaluation where they had the second lowest scores. They had an average of 74 (SD = 61) employees and most commonly represented the services (45%) and health care and social assistance (35%) industries.

#### The business strategy-focused profile

Just over one-quarter (27%) of businesses in the sample were in the business strategy-focused profile. This profile name was chosen because businesses generally had higher scores on the benchmarks, but lower scores on the leadership and climate variables. Businesses had the second highest scores on organizational supports, worksite assessment, health policies and programs, safety policies and programs, engagement, and evaluation, but they had the second lowest scores on leadership commitment to safety, leadership commitment to health, safety climate, and health climate. Businesses in this profile had on average 117 employees (SD = 127) and most commonly represented the services (23%), health care and social assistance (23%), and public administration (19%) industries.

#### The culture-focused profile

Businesses in the culture-focused profile represented 31% of all businesses. This profile name was chosen because businesses generally had higher scores on the leadership and climate variables, but lower scores on the benchmarks. Businesses in this profile had the second highest scores on leadership commitment to safety, leadership commitment to health, safety climate, and health climate, but either the lowest or second lowest scores on organizational supports, worksite assessment, health policies and programs, safety policies and programs, engagement, and evaluation. The businesses had an average of 49 employees (SD = 61). The most common industries represented in this profile were services (37%), health care and social assistance (17%), and manufacturing (17%).

#### The advanced profile

There were few businesses in the advanced profile (9%). Businesses in this profile had the highest scores on all indicators. They had on average 89 employees (SD = 73) and were more diverse in terms of industries. The most common industries represented services (22%), health care and social assistance (22%), and 11% each in construction, manufacturing, mining and oil, public administration, and retail/wholesale trade.

### TWH profile relationship to employee demographics and behavior

There were few differences in profiles by employee demographics (see Table 3). There was a noticeably higher proportion of females in businesses with the beginner profile, compared to all other profiles. Businesses in the culture-focused or advanced profiles were more diverse in terms of race, but less diverse in terms of ethnicity. There were more parttime employees in the beginner profile than the other profiles. Employees in each of the profiles differed on many of the other demographic variables, but there were few practical differences.

**Table 3:** Demographic characteristics of employee sample by final latent profile solution (*n* = 2868)

	Profile 1: Beginner	Profile 2: Business strategy- focused	Profile 3: Culture- focused	Profile 4: Advanced
Age	42 (13)	43 (13)	39 (13)	39 (13)
Gender				
Male	199 (22%)	313 (38%)	188 (37%)	120 (45%)
Female	695 (77%)	507 (61%)	313 (62%)	141 (54%)
Other	5 (1%)	6 (1%)	5 (1%)	0 (0%)
Race				
White	775 (92%)	761 (95%)	445 (89%)	245 (84%)
Black or African American	20 (2%)	12 (2%)	24 (5%)	6 (2%)
Asian	17 (2%)	14 (2%)	7 (1%)	4 (2%)
Native American or Alaskan Native	24 (3%)	12 (2%)	21 (4%)	5 (2%)
Native Hawaiian or Other Pacific Islander	9 (1%)	6 (1%)	1 (0%)	0 (0%)
Ethnicity				
Hispanic or Latino or Spanish Origin	162 (18%)	94 (11%)	80 (16%)	14 (5%)
Not Hispanic or Latino or Spanish Origin	731 (82%)	731 (87%)	424 (84%)	245 (95%)
Education				
Did not complete high school	3 (0%)	5 (1%)	10 (2%)	3 (1%)
High school diploma or GED	73 (9%)	91 (13%)	76 (17%)	23 (10%)
Some college or 2-year degree	175 (22%)	195 (27%)	148 (32%)	55 (24%)
4-year college degree	291 (37%)	291 (40%)	176 (29%)	118 (51%)
Graduate or professional degree	236 (30%)	145 (20%)	47 (10%)	33 (14%)
Job Level				
Supervisor	517 (57%)	483 (58%)	267 (53%)	134 (51%)
Non-supervisor	383 (43%)	345 (42%)	240 (47%)	127 (49%)
Job Tenure (years)	6 (6)	6 (8)	5 (6)	3 (3)
Household income				
< $50,000	276 (36%)	206 (29%)	174 (39%)	66 (29%)
$50,001 - $100,000	299 (39%)	323 (45%)	161 (36%)	77 (34%)
> $100,000	195 (25%)	189 (26%)	117 (26%)	85 (37%)
Type of Work				
Full-time	762 (85%)	750 (91%)	436 (86%)	231 (89%)
Part-time	131 (15%)	72 (9%)	72 (14%)	30 (12%)
Work hours per week	38 (12)	41 (10)	39 (12)	42 (12)
Payment scheme				
Salary	482 (54%)	380 (46%)	201 (40%)	139 (54%)
Hourly	419 (47%)	445 (54%)	304 (60%)	120 (46%)
Contractor or consultant	27 (3%)	20 (2%)	28 (6%)	15 (6%)
Shift work	154 (17%)	107 (13%)	105 (21%)	47 (18%)

The results of the linear regression are presented in Table 4. Employees working in a business strategy-focused, culture-focused, and advanced profiles all had significantly better behaviors than employees working for a business with the beginner profile. Compared to employees working for a business in the beginner profile, employee health behaviors were best when they worked for a business with the advanced profile, but employee safety behaviors were best when they worked for a business with either the culture-focused or advanced profile. A post-hoc pairwise comparison of effects between all combinations of profiles using the Bonferroni method of adjusting for multiple comparisons revealed that the there were minimal differences between each of the three non-beginner profiles (see Additional file 1).

**Table 4:** Relationship between business TWH profiles and employee safety and health behaviors (*n* = 2868)

	Linear regression				Adjusted average behavior score by profile			
	coefficient (95% CI)				M (95% CI)			
	Profile 1: Beginner (ref)	Profile 2: Business strategy-focus	Profile 3: Culture- focus	Profile 4: Advanced	Profile 1: Beginner	Profile 2: Business strategy-focus	Profile 3: Culture-focus	Profile 4: Advanced
Safety behaviors	1.00 (ref)	0.12 (−0.01, 0.24)*	0.24 (0.10, 0.37)***	0.22 (0.04, 0.40)**	3.70 (3.61, 3.79)	3.82 (3.73, 3.90)	3.94 (3.83, 4.04)	3.92 (3.76, 4.08)
Health behaviors	1.00 (ref)	0.21 (0.08, 0.35)***	0.33 (0.18, 0.47)***	0.44 (0.24, 0.63)***	3.34 (3.25, 3.46)	3.56 (3.46, 3.65)	3.67 (3.56, 3.78)	3.77 (3.16, 3.95)

***p < 0.01, **p < 0.05, *p < 0.10

The linear regression models controlled for industry, number of employees, whether they were in a management role, tenure, age, and gender. The models also included a random intercept for businesses to account for variability in the outcome by business. The sample size for the safety behaviors model was 2213 and for the health behaviors it was 2215

## Discussion

Businesses often report that they have difficulty meeting their engagement goals for their workplace safety and worker well-being programs. They often respond in a transactional way –adopting additional programs and policies. The present study demonstrates that employee participation in health and safety behaviors is greater when organizations display strong leadership commitment to employee health and safety and when they are perceived by employees as having climates supportive of health and safety practices. Our findings suggest that workers’ health and safety actions are strongest in those workplaces that have focused on the combination of not only its TWH policies and programs, but also on establishing leadership that establishes a climate that supports and encourages health and safety behaviors.

The results of the present study add to the occupational health and safety small business literature by identifying four ways in which small business address TWH. As we might expect, few small businesses have both the TWH business strategies and the leadership and climate to support the strategies. Indeed, prior research demonstrates that smaller businesses lag in their efforts to implement TWH policies and practices [7]. However, about half of the businesses in our study either had stronger TWH business strategies or stronger leadership and climate (i.e., culture-focused). This suggests there is variation in small business approaches to TWH. These findings have significant implications for how we design small business TWH interventions.

As hypothesized, we found that employees working for businesses with advanced TWH practices reported better safety and health behaviors than did employees working for businesses with less advanced TWH practices. This is consistent with prior research demonstrating the relationship between safety and health leadership, climate, and employee behavior [4, 5]. There is some evidence that safety management practices are associated with better safety behaviors and lower injury rates (e.g., [26, 27]). Similarly, worksite wellness practices are associated with better employee engagement and health risk factors (e.g, [28]). There is emerging TWH intervention research suggesting that a focus on policy and program development and implementation via management and leadership practices lead to changes in employee practices and lifestyle health risks (e.g., [29]).

Employee engagement in TWH is critical to the success of any TWH initiative [11, 30], given the priority that TWH frameworks place on workplace safety, it is encouraging to see that regardless of which TWH programs and policies are in place, engagement in safety behaviors is highest when businesses focus on their culture. Prior research focused on the relationships between leadership, climate, and behavior without including more context around TWH business strategies [8]. Our study adds to this literature by supporting the primacy of leadership and climate in eliciting employee engagement. Businesses with a business strategy-focused profile with better scores on benchmarks and worse scores on leadership and climate elicited better employee health and safety behaviors than did businesses that were just beginning to develop their methods for TWH in the beginner profile with lowest benchmark, leadership and climate scores. However, given prior literature, it is not surprising to observe that organizations in the two business profiles that are focused on culture elicited the best employee health and safety behaviors [4, 31].

Our findings are also similar to prior workplace health protection and promotion research using a latent profile analysis approach. Biswas et al. [22] profiled businesses based on the co-occurrence of business health protection and promotion practices (e.g., safety audits and flexible work hours). They found that larger businesses (> 500 employees) were more likely to be in the highest co-occurrence profile than were small businesses (< 100 employees). We cannot directly compare our results to theirs as we did not have employers with more than 500 employees in our study, but we did find that, on average, the largest employers in our study fell into the profile with more TWH policies and programs (i.e., business strategy profile) and the smallest organizations fell into a profile with fewer TWH policies and programs (i.e., culture-focused profile). Weaver et al. [21] characterized businesses using a workplace health promotion checklist and found that some businesses fell into a profile characterized by a supportive environment but little operational initiatives, which is similar to our culture-focused profile. Finally, we found that those businesses that fit the profiles with more positive leadership and climate for health and safety employed workers who reported better health and safety behaviors. Similarly, Klug et al. [19] found that positive workplace healthy leadership profiles were related to self-rated health.

### Future research

There are a number of important priorities for future research in this area. First, longitudinal data are needed to understand how TWH profiles in small businesses change over time. Additionally, this research can inform whether contextual factors such as age of business, size of business, industry, and geographical region influence change in profile. Second, in light of the COVID-19 pandemic, it would be informative to understand which profile(s) are associated with the best employee health outcomes during emergencies. We recently observed that employees’ self-reported well-being is highest in those small businesses which had stronger health and safety climates at the start of the COVID-19 pandemic [32]. Thus, we might expect that businesses with a business culture-focused or advanced profile would elicit the best employee health outcomes during emergencies. Third, research is needed to understand how TWH interventions help small businesses adopt TWH practices and improve their profile. We hypothesize that TWH leadership training is an important intervention that helps small businesses achieve an advanced profile [2]. In a study of 38 leaders from 23 small businesses, we found that a TWH leadership training program helped small business leaders develop their TWH leadership practices in the context of their business’s TWH strategy [33]. Current work is being conducted to evaluate how this training can help small businesses improve their TWH profiles via our broader Small+Safe+Well study [2]. Small businesses in that study are randomized to receive either an intervention that was intended to help them change their TWH policies and programs or that intervention plus a TWH leadership training. Our aim is to help the small business undergo organizational change by modifying TWH policies and programs, leadership, and climate, with the ultimate goal of helping its employees improve their safety, health and well-being.

### Public health practice implications

Public health practitioners may use assessments such as those used in the present study to determine current TWH approaches in small businesses and identify ways in which to help them improve. Consistent with prior research on the paucity of protections for workforce safety and health promotion programs [6, 7, 34], we observed that some businesses were at a *beginner* stage with the lowest scores on all indicators of TWH. Our research adds to this literature by showing that some small businesses had employees reporting positive perceptions of safety and health leadership and climate (i.e., culture-focused). These small businesses may be at an advantage when it comes to developing and implementing a TWH business strategy because they already have a supportive environment [35]. However, we hypothesize that without the commensurate TWH business strategies, these small businesses may find themselves in an *unsustainable* situation over time where there are few methods to keep workers healthy and safe. In this case, the small business may only need assistance developing a TWH business strategy that can help them cope with changing threats to worker safety and health. On the other hand, we observed that some businesses have invested in TWH business strategies but without ensuring adequate employee perceived organizational commitment to health and safety. We hypothesize that they may find themselves in an *ineffective* situation over time where methods to keep workers healthy and safe are rarely used in practice [4, 31]. In this case, small businesses may benefit from TWH interventions that focus on leadership.

### Strengths and weaknesses

Our study has a few strengths and weaknesses. A strength of this study is the variety of businesses from multiple industries and from both urban and rural settings represented in the study. We also had access to a unique data source with information from both the perspective of the business as well as the employee. Furthermore, the variables gather from both the Healthy Workplace Assessment and the Employee Health and Safety Culture survey have been previously evaluated for reliability and validity [7, 8]. However, we did not have access to information that would describe the quality of TWH business strategies. Another limitation is the potential for misclassification of business into a profile. However, the average latent profile probabilities for most likely latent profile membership were high (> 0.90) indicating that the potential for misclassification is low. Finally, our cross-section data limits our ability to make causal claims about the relationship between the profiles and employee safety and health behaviors.

## Conclusion

Our study demonstrates that employee engagement in TWH is associated with TWH business practices that focus on having a business strategy for how they implement a TWH approach as well as leadership commitment to these strategies and having an environment that fosters positive safety and health climates. From a public health practice standpoint, our data suggest that TWH assessments should be used to identify what types of TWH interventions will most benefit small businesses. More research is needed to understand whether small businesses can improve upon their profile overtime.

## Supplementary Information

**Additional file 1: Table 1.** Inter-rater reliability (ICC (1)) and inter-rater agreement (r*wgj) statistics used to justify aggregation of responses to the business-level (*n* = 2868). **Table 2.** Means, standard deviations, and correlations of indicators of TWH (*n* = 97). **Table 3.** Fit statistics of latent profile analyses evaluating 2 to 4 profile solutions (*n =* 97). **Table 4.** Pairwise comparisons between profiles for safety behavior outcome. **Table 5.** Pairwise comparisons between profiles for health behavior outcome.

## Data Availability

The de-identified datasets used and/or analyzed during the current study are available from the corresponding author on reasonable request.
