# Supplementary material for: Profiles of total worker health® in United States small businesses
Source: BMC Public Health. 2021 May 29;21:1010. doi: 10.1186/s12889-021-11045-8 (PMC8164062; doi:10.1186/s12889-021-11045-8)
Supplement: Supplementary file 1 — Additional file 1: Table 1. Inter-rater reliability (ICC (1)) and inter-rater agreement (r*wgj) statistics used to justify aggregation of responses to the business-level (n = 2868). Table 2. Means, standard deviations, and correlations of indicators of TWH (n = 97). Table 3. Fit statistics of latent profile analyses evaluating 2 to 4 profile solutions (n = 97). Table 4. Pairwise comparisons between profiles for safety behavior outcome. Table 5. Pairwise comparisons between profiles for health behavior outcome. [file 12889_2021_11045_MOESM1_ESM.docx]

**Profiles of Total Worker Health® in United States small businesses**

**Additional File 1**

| **Table 1** Inter-rater reliability (ICC(1)) and inter-rater agreement (r*wgj) statistics used to justify aggregation of responses to the business-level (n = 2,868) | | |
| --- | --- | --- |
|  | ICC (1) | r*wgj |
|  | Estimate (95% CI) | Estimate |
| Leadership commitment to safety | 0.12 (0.08, 0.17) | 0.84 |
| Leadership commitment to health | 0.12 (0.08, 0.17) | 0.82 |
| Safety climate | 0.17 (0.12, 0.23) | 0.87 |
| Health climate | 0.12 (0.08, 0.18) | 0.84 |

| **Table 2** Means, standard deviations, and correlations of indicators of TWH (n=97) | | | | | | | | | | | | | |
| --- | --- | --- | --- | --- | --- | --- | --- | --- | --- | --- | --- | --- | --- |
|  |  | Mean | SD | 1 | 2 | 3 | 4 | 5 | 6 | 7 | 8 | 9 | 10 |
| 1 | Organizational supports | 15.41 | 6.53 | 1 |  |  |  |  |  |  |  |  |  |
| 2 | Worksite assessment | 2.86 | 2.81 | 0.63*** | 1 |  |  |  |  |  |  |  |  |
| 3 | Health policies & programs | 3.40 | 2.89 | 0.55*** | 0.39*** | 1 |  |  |  |  |  |  |  |
| 4 | Safety policies & programs | 9.54 | 3.68 | 0.43*** | 0.55*** | 0.40*** | 1 |  |  |  |  |  |  |
| 5 | Engagement | 7.05 | 2.58 | 0.61*** | 0.57*** | 0.55*** | 0.45*** | 1 |  |  |  |  |  |
| 6 | Evaluation | 2.97 | 1.87 | 0.51*** | 0.54*** | 0.54*** | 0.48*** | 0.53*** | 1 |  |  |  |  |
| 7 | Leadership commitment to safety | 3.65 | 0.40 | 0.24* | 0.247* | -0.01 | 0.17 | 0.19 | 0.16 | 1 |  |  |  |
| 8 | Leadership commitment to health | 3.57 | 0.44 | 0.21* | 0.09 | 0.20* | -0.12 | 0.22* | 0.14 | 0.61*** | 1 |  |  |
| 9 | Safety climate | 3.92 | 0.41 | 0.21* | 0.16 | 0.21* | -0.11 | 0.22* | 0.15 | 0.57*** | 0.88*** | 1 |  |
| 10 | Health climate | 3.79 | 0.41 | 0.30** | 0.35*** | 0.02 | 0.32** | 0.21* | 0.18 | 0.88*** | 0.46*** | 0.53*** | 1 |

| **Table 3** Fit statistics of latent profile analyses evaluating 2 to 4 profile solutions (n=97) | | | |
| --- | --- | --- | --- |
|  | 2 profile solution | 3 profile solution | 4 profile solution |
| AIC | 3200.97 | 3118.84 | 3084.66 |
| BIC | 3280.79 | 3226.98 | 3221.12 |
| Sample Size Adjusted BIC | 3182.9 | 3094.36 | 3053.77 |
| Entropy | 0.93 | 0.88 | 0.89 |
| Adjusted Likelihood Ratio Test (df; p-value) | 214.83 (11; 0.80) | 102.10 (11; 0.47) | 55.08 (11;0.39) |
| Bootstrapped Likelihood Ratio Test (df; p-value) | 219.10 (11; 0.00) | 104.13 (11; 0.00) | 56.18 (11; 0.00) |
| Final profile counts for the latent profile based on their most likely latent profile membership |  |  |  |
| profile 1 | 72 | 32 | 32 |
| profile 2 | 25 | 24 | 26 |
| profile 3 |  | 41 | 30 |
| profile 4 |  |  | 9 |
| Average latent profile probabilities for most likely latent profile membership |  |  |  |
| profile 1 | 0.98 | 0.96 | 0.94 |
| profile 2 | 0.96 | 0.96 | 0.95 |
| profile 3 |  | 0.93 | 0.93 |
| profile 4 |  |  | 0.96 |

| **Table 4** Pairwise comparisons between profiles for safety behavior outcome | | | | |
| --- | --- | --- | --- | --- |
|  | Contrast | SE | Bonferroni 95% CI | |
| 2 vs 1 | 0.11 | 0.06 | -0.06 | 0.28 |
| 3 vs 1 | 0.23 | 0.07 | 0.05 | 0.41 |
| 4 vs 1 | 0.22 | 0.09 | -0.03 | 0.47 |
| 3 vs 2 | 0.12 | 0.07 | -0.07 | 0.31 |
| 4 vs 2 | 0.11 | 0.09 | -0.13 | 0.35 |
| 4 vs 3 | -0.01 | 0.10 | -0.26 | 0.24 |

| **Table 5** Pairwise comparisons between profiles for health behavior outcome | | | | |
| --- | --- | --- | --- | --- |
|  | Contrast | SE | Bonferroni 95% CI | |
| 2 vs 1 | 0.21 | 0.07 | 0.03 | 0.39 |
| 3 vs 1 | 0.33 | 0.07 | 0.13 | 0.52 |
| 4 vs 1 | 0.43 | 0.10 | 0.17 | 0.70 |
| 3 vs 2 | 0.12 | 0.08 | -0.08 | 0.32 |
| 4 vs 2 | 0.22 | 0.10 | -0.03 | 0.48 |
| 4 vs 3 | 0.11 | 0.10 | -0.16 | 0.37 |
